# Hemophagocytic lymphohistiocytosis complicating a T-cell rich B-cell lymphoma

**DOI:** 10.1186/s12878-016-0065-5

**Published:** 2016-11-24

**Authors:** El Mehdi Mahtat, Maryem Zine, Mohamed Allaoui, Malika Kerbout, Nezha Messaoudi, Kamal Doghmi, Mohamed Mikdame

**Affiliations:** 1 Service d’Hématologie Clinique, Hôpital Militaire d’Instruction Mohamed V, Rabat, Morocco; 2 Laboratoire d’Anatomie Pathologique, Hôpital Militaire d’Instruction Mohamed V, Rabat, Morocco

## Abstract

**Background:**

Hemophagocytic lymphohistiocytosis in adults is often secundary to an infection or a neoplasm. In this last case, T cell lymphomas are the most frequent causes. Hemophagocytic lymphohistiocytosis secundary to a B cell lymphoma has been rarely reported.

**Case presentation:**

We describe a case of a hemophagocytic lymphohistiocytosis complicating a T-cell rich B-cell lymphoma treated with conventionnal chemotherapy leading to a complete remission.

**Conclusion:**

Prompt etiologic diagnosis and treatment of hemophagocytic lymphohistiocytosis leads to satisfactory outcome.

## Background

Hemophagocytic lymphohistiocytosis (HLH) is a rare and often fatal inflammatory disease. It is either primary or secondary to inflammatory diseases, infections or malignancies. In the latter case, T phenotype non-Hodgkin lymphoma (NHL) is the most common cause [1]. The association with B lymphomas is rare [2]. In this situation, lymphoma chemotherapy treatment should be initiated promptly to control HLH. T-cell rich B-cell lymphoma is a rare entity representing 1 to 3 % of diffuse large B-cell NHL [3].

We describe in this paper the case of a patient with a hemophagocytic lymphohistiocytosis revealing a T cells rich B-cell NHL.

## Case presentation

A 52-year-old male patient without any significant medical history was admitted to our department for febrile bicytopenia. He reported an anemic syndrome as he had been complaining of fatigue and exertional dyspnea for 8 months before his admission. Fever and significant weight loss were also reported over the last month before his admission.

Physical examination revealed fever (39.8 °), pallor, splenomegaly (4 cm below left costal margin), right axillary and bilateral inguinal lymphadenopathies (the most voluminous measured 3 cm of diameter).

Laboratory tests found haemoglobin level at 70 g/L (range 130–165 G/L) with a mean corpuscular volume of 80 fl (range 80–96 fl), leukocytes at 2.9 G/L (range 4–10 G/L) (neutrophils 1.5 G/L and lymphocytes 0.9 G/L). Platelets were 39 G/L (range 150–400 G/L). Reticulocyte count was 43 G/L (50–120 G/L). Biochemical tests showed an increased LDH rate at 508 IU/L (upper limit: 192 UI/L) and serum ferritin at 4456 ng/mL (range 23–336 ng/mL). Triglycerides were 225 mg/dL (range 101–150 mg/dL). Fibrinogen was also raised at 6,72 g/L (range 1,5–4 g/L). Infectious tests, including EBV PCR screening, were negative.

**Fig. 1:**
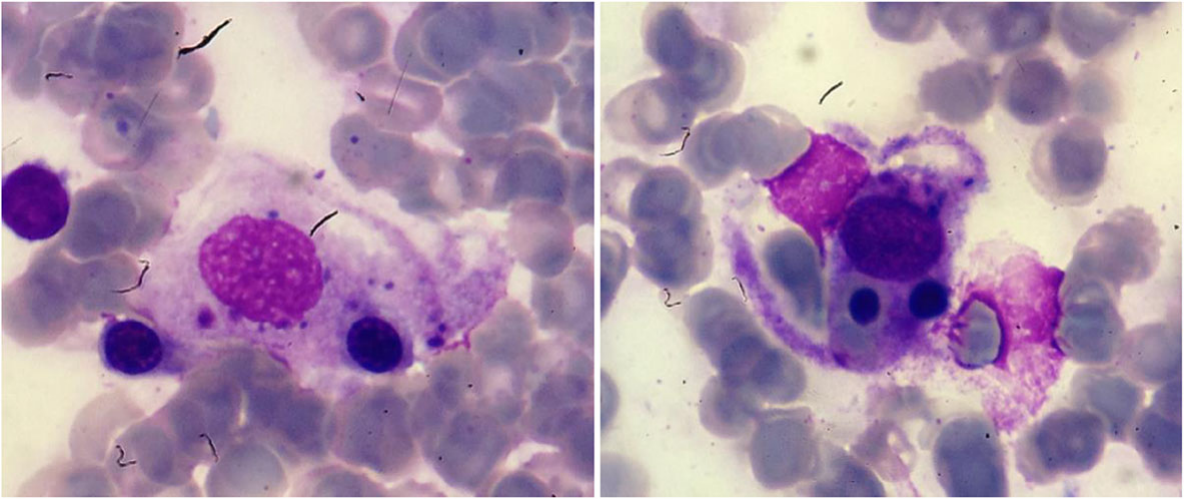
Marrow aspirate smear showing examples of hemophagocytosis, May Grunwald Giemsa stain, × 1000

Hemophagocytic lymphohistiocytosis was strongly suspected according to Henter criteria [4] and a bone marrow aspiration was performed showing a rich marrow with hemophagocytosis (Fig. 1) without lymphomatous infiltration. Thus, the diagnosis of hemophagocytic lymphohistiocytosis was confirmed.

The patient received a pulse of steroids (methylprednisolone 25 mg/kg/day for 3 days followed by prednisolone 2 mg/kg/day) as well as red blood cells and platelets supportive transfusion.

**Fig. 2:**
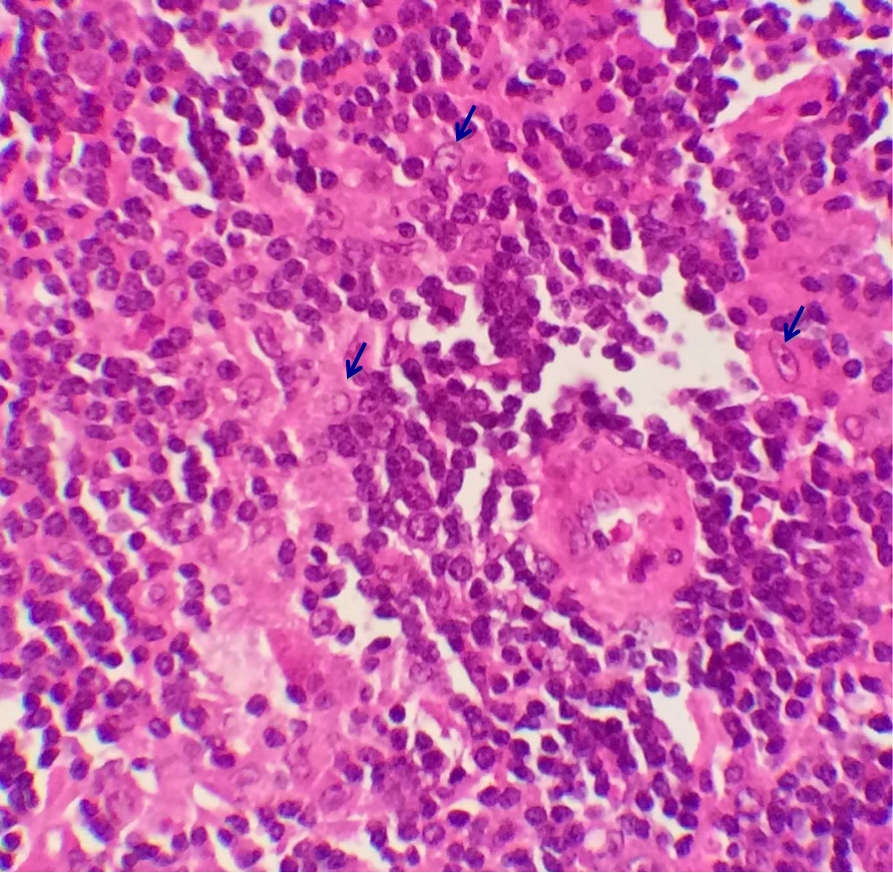
Lymphadenopathy biopsy showing diffuse lymphohystiocytic infiltration the normal architecture with scattered large atypical cells (*Arrows*), H&E stain, × 400

**Fig. 3:**
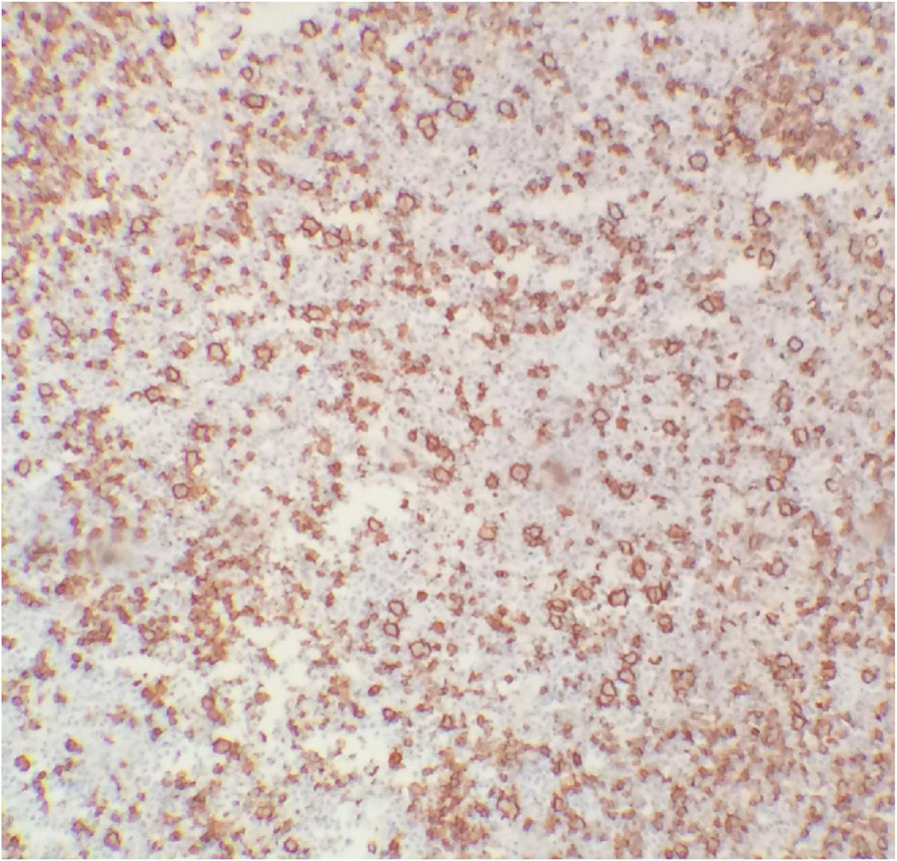
CD20 immunostain highlights large neoplastic B cells, × 400

**Fig. 4:**
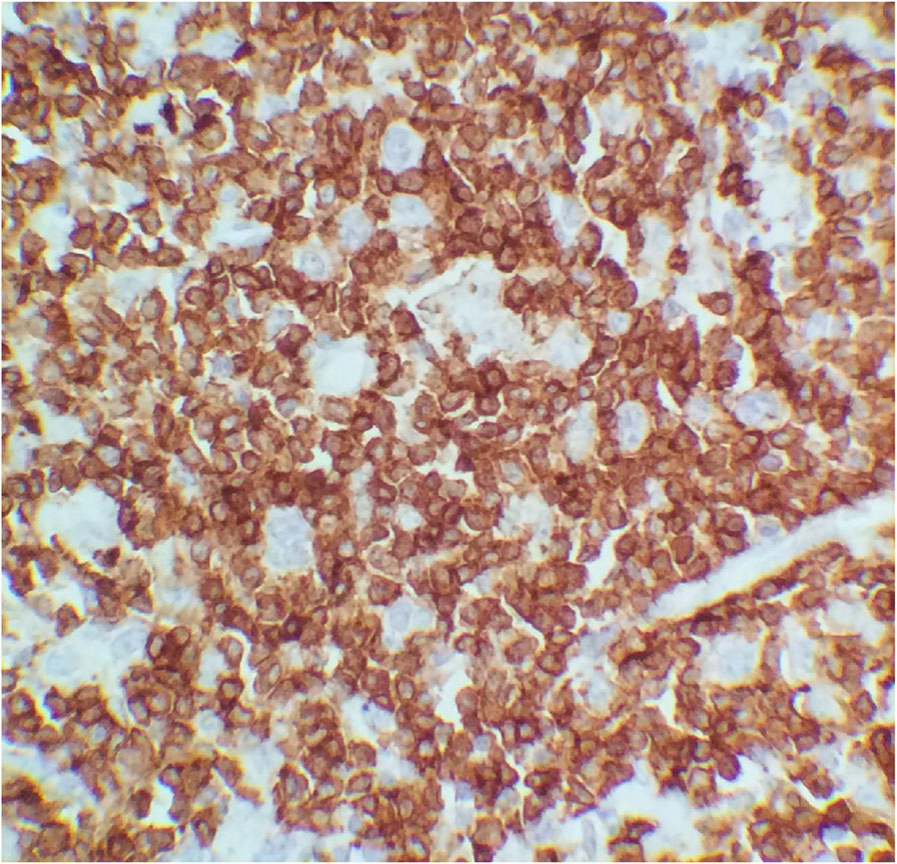
CD3 immunostain marking small lymphocytes × 1000

A biopsy of axillary lymphadenopathy showed a lymph node parenchyma which overall architecture is erased by diffuse immunoblastic large cells proliferation. These scattered large neoplastic cells are present on a background rich in histiocytes and small lymphocytes (Fig. 2). Immunohistochemistry studies showed expression of CD20 in the large neoplastic cells (Fig. 3) and CD3 in the small T cells (Fig. 4). CD30, CD15 and EBV stains were negative.

**Fig. 5:**
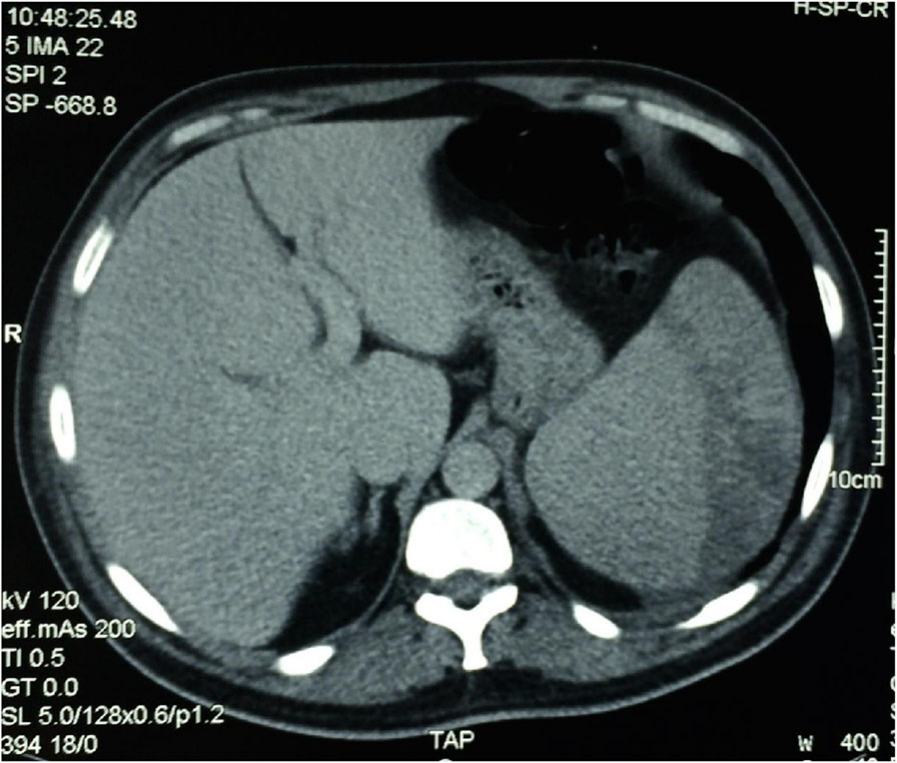
CT scan abdomen showing a 20 cm large spleen with multiple hypodensities (infarcts)

Computed tomography (CT) scan of the chest, abdomen and pelvis showed enlarged lymph nodes on both sides of diaphragm and a 20 cm large spleen with multiple hypodensities, likely to be related to infarcts (Fig. 5). A bone marrow biopsy was also performed and showed no infiltration.

Therefore, this case was diagnosed as T-cell rich B-cell Hodgkin lymphoma stage III B (Ann Arbor staging) complicated by a HLH.

The patient was treated with chemotherapy combining rituximab, cyclophosphamide, doxorubicin, vincristine and prednisone (R-CHOP). He received eight 21-day cycles associated to 4 injections of prophylactic intrathecal chemotherapy (methotrexate, cytarabine, methylprednisolone). The interim and the end of treatment revaluations showed complete remission with normalization of initially abnormal biological parameters.

After 10 months of follow-up, the patient presented with axillar lymph nodes without general symptoms. The biopsy of the lymph nodes showed the same aspect as at the diagnosis. The patient is now undergoing salvage therapy by Rituximab, dexamethasone, ifosfamide, carboplatin and etoposide (R-DICE) regimen. It will be followed by intensification and autologous stem cell transplantation.

## Discussion

HLH is the result of a secondary immune response to stimuli which regulation is no longer controlled by the natural killer lymphocytes (NK-L) [5]. In familiar cases, the major mutations affect granule-mediated cytotoxicity pathways. The cytotoxicity defect of the NK lymphocytes is the main pathophysiological signature of HLH [6]. This promotes proliferation and continuous activation of antigen presenting cells with a hyper-secretion of cytokines and chemokines, causing a “cytokine storm” [7]. Indeed, activated T lymphocytes (TL) secrete interferon gamma in large amounts inducing expansion and activation of CD8 T cells, histiocytes and macrophages. These cells infiltrate various organs, including the hematopoïetic organs [1]. The “cytokine storm” is responsible for clinical features and laboratory findings of multi-organ failure as seen in the HLH. Interleukin (IL) 1, IL-6 and tumor necrosis factor alpha are responsible for fever. Hypertriglyceridemia is secondary to the inhibition of lipoprotein lipase and stimulation of the synthesis of triglycerides by INFγ et TNFa [8]. These cytokines also inhibit normal hematopoiesis inducing cytopenias. Hyperferritinaemia and hypofibrinogenemia are secondary to the continuous activation of macrophages [5].

The diagnosis of HLH is based on the combination of clinical and laboratory criteria (Table 1) [4]. Clinicians must think about it in the case of fever of unknown origin. This syndrome can be hereditary or acquired. In the latter case, it is most often associated with infections (49 %); infection with Epstein Barr Virus (EBV) being the most common cause. It is secondary to neoplasia in up to 27 % of cases and associated with rheumatic diseases in 7 % and immunodeficiencies in 6 % of cases [5]. When HLH is secondary to malignancies, it is most often associated with T or NK phenotype lymphoma or leukemia. However associations to anaplastic lymphomas, acute B lineage lymphoblastic or myeloblastic leukemias, as well as solid tumors have been reported [5]. The association with NHL phenotype B is rarely reported; often described in older patients with less direct involvement of bone marrow in contrast to T lymphoma secondary HLH [2]. Indeed, our patient had no bone marrow infiltration. In the case HLH in adults, a thoracic and abdominal CT scan and a bone marrow biopsy looking for a lymphomatous infiltration can be helpful to find a possible underlying malignancy [1]. The peculiarity of our case is the association of HLH to a particular histological presentation of B-cell NHL. The T-cell rich B-cell lymphoma is a rare histological form accounting for 1–3 % of DLBCL. Histologically, it is characterized by the presence of less than 10 % large B cells in a cellular background made of small cytotoxic lymphocytes and histiocytes [3]. The main differential diagnosis of this entity is nodular lymphocyte-predominant Hodgkin lymphoma. Immunohistochemistry can rule out this diagnosis with the negativity of CD30 and CD15 on large cells, and a clear-cut CD20 positivity on the large cells [3]. The combination of a T-cell rich B-cell lymphoma and HLH has already been described (Table 2). Mitterer et al reported a case of T cells rich B-cell NHL (TCRBCL) with HLH and concomitant EBV reactivation, but the malignant cells did not express EBV oncoprotein LMP-1 and the EBV infection was probably related to the immunodeficiency induced by the HLH syndrome in that case [9]. In our case there was no evidence of ongoing EBV infection. The link between HLH and TCRBCL is not accidental, in fact the study of the gene expression profile of T-cell rich B-cell lymphoma showed tolerogenic immune response signatures of the host explaining the aggressive nature of this type of lymphoma and the associated immune reactions [10]. It was also demonstrated that the immunomodulatory molecule programmed death ligand 1 (PD-L1) is expressed by the tumor cells and the histiocytes in T-cell rich B NHL and may inhibit T-cell immunity [11].

**Table 1:** Diagnostic criteria for HLH [4]

≥ five of the eight criteria listed below:	
Fever	
Splenomegaly	
Cytopenias (affecting at least two of three lineages in the peripheral blood): • Hemoglobin < 9 g/dl • Platelets < 100 G/mm3 • Neutrophils <1 G/mm3	
Hypertriglyceridemia (fasting, 265 mg/100 ml) and/or hypofibrinogenemia (150 mg/100 ml)	
Hemophagocytosis in bone marrow, spleen or lymph nodes	
Ferritin ≥ 500 ng/ml	
Low or absent Natural Killer cell activity	
Soluble IL-2 receptor ≥ 2400 U/ml

**Table 2:** Clinical, biological, therapeutic and evolution features of previously reported cases of TCRBCL associated HLH

Case	Sex	Age	Clinical features	Laboratory findings	Pathology	EBV	Treatment	Outcome
*Mitterrer* et al. [9]	Female	30	B symptoms, splenomegaly	Moderate pancytopenia, high LDH	Spleen: hemophagocytosis Hepatic nodules: TCRBCL	Reactivated EBV infection serological profile	MOPP-ABV then high dose methotrexate, vincristine and etoposide followed by AHSCT	Sustained CR for 2 years
*Devitt* et al. [12]	Male	30	Fever, jaundice, B symptoms, splenomegaly, repiratory failure	Hyperferritinemia Pancytopenia Hyperbilirubinemia Elevated liver enzymes High LDH	Bone marrow: Hemophagocytosis and lymphomatous infiltration	Negative (in situ hybridization)	HLH 2004, R-EPOCH	CR followed by AHSCT
*Aljitawi et Boone* [13]	Male	34	Relapse of previously treated TCRBCL Fever, jaundice, hepatosplenomegaly, ascites	Hyperferritinemia Pancytopenia Hyperbilirubinemia High soluble IL2-R	Bone marrow: Hemphagocytosis, relapsed TCRBCL	NA	Salvage therapy (NA)	Relapse after months and death
*Jiang* et al. [14]	Male	20	Jaundice Fatigue Abdominal disconfort Fever	Acute hepatitis Pancytopenia Hyperferritinemia High soluble IL2-R	Bone marrow and lymph node: TCRBCL	NA	R-CHOP	CR
*Our case*	Male	52	B symptoms, splenomegaly Lymph nodes	Pancytopenia High LDH Hyperferritinemia	Bone marrow: hemophagocytosis Lymph node: TCRBCL	Negative (biopsy and peripheral blood PCR)	R-CHOP	Relapse after 10 months

*LDH* lacticodeshydrogenase, *R-EPOCH* Rituximab, etoposide, prednisone, vincristine, cyclophosphamide and doxorubicine, *CR* complete remission, *AHSCT* autologous hematopoietic stem cell transplantation, *TCRBCL* T-cell rich B-cell lymphoma, *MOPP-ABV* mechlorethamine, vincristine, procarbazine, prednisone/doxorubicin, bleomycin, vincristine, *PCR* polymerase chain reaction

## Conclusion

Hemophagocytic lymphohistiocytosis is a diagnostic and therapeutic emergency. The main underlying causes of this syndrome in adults are either infectious or T lymphomatous proliferations. The association with T cells rich B lymphoma is rarely described. A prompt antilymphomatous chemotherapy should be initiated to control the life-threatening HLH.
